# Surgical Retrieval of Tooth Fragment from Lower Lip and Reattachment after 6 Months of Trauma

**DOI:** 10.5005/jp-journals-10005-1302

**Published:** 2015-08-11

**Authors:** Mohita Marwaha, Kalpana Bansal, Ankit Srivastava, Neha Maheshwari

**Affiliations:** Associate Professor,Department of Pedodontics and Preventive Dentistry, SGT Dental College and Research Institute, Gurgaon, Haryana India; Professor and Head, Department of Pedodontics and Preventive Dentistry, SGT Dental College and Research Institute, Gurgaon, Haryana India; Senior Lecturer, Department of Pedodontics and Preventive Dentistry, SGT Dental College and Research Institute, Gurgaon, Haryana India; Postgraduate Student, Department of Pedodontics and Preventive Dentistry, SGT Dental College and Research Institute, Gurgaon, Haryana India

**Keywords:** Children, Maxillary central incisor, Trauma.

## Abstract

Dental traumas are one of the most frequent facial traumas especially in children. Maxillary incisors are the most frequently involved teeth. Here we present, a report of a child who sustained a crown fracture with lost portion of tooth embedded in her lower lip for 6 months. The fragment was surgically retrieved and successfully reattached to the fractured 21 using acid-etch resin technique.

**How to cite this article:** Marwaha M, Bansal K, Srivastava A, Maheshwari N. Surgical Retrieval of Tooth Fragment from Lower Lip and Reattachment after 6 Months of Trauma. Int J Clin Pediatr Dent 2015;8(2):145-148.

## INTRODUCTION

Trauma to teeth is a common situation in a pediatric patient. Every dental professional must be prepared to assess and treat when necessary. It not only damages the dentition but also affect the patient psychologically.^1^ The teeth that are most commonly involved in trauma are the maxillary central incisors as they occupy a more vulnerable position in the arch. The reported percentages of simple and complex coronal fractures in children due to trauma are 28 to 44% and 11 to 15% respectively.^2^ Most dental injuries occur between 2 and 3 years and between 8 and 12 years of age. They are more common in boys because of their active involvement in extracurricular activities.^3,4^ A number of techniques have been developed to restore the fractured crown. Early techniques include: stainless steel crowns, basket crowns, orthodontic bands, pin retained resin, porcelain bonded crown and composite resin.^5,6^ The first case of reattaching a fractured incisor fragment was reported in 1964 by a pediatric dentist at Hebrew University, Hadassah School of Dentistry.^7^ Tennery (1978)^8^ was the first to report the reattachment of a fractured fragment using acid etch technique. The introduction of composite restorative materials in combination with the use of the acid-etch technique to bond composite to enamel, made possible the restoration of the fractured incisor with little or no additional tooth preparation.^6,7^

Reattachment of tooth fragments should be the first choice and is a viable alternative to conventional approach because of simplicity, natural esthetics, conservation of tooth structure.

Among the advantages of reattachment are^9^: Good esthetics, color match to the remaining crown portion, preservation of incisal translucency; conservation, maintenance of original tooth contours, preservation of adequate occlusal contacts; were similar to adjacent/ opposed tooth; financial and economic aspects of a conservative, one-visit treatment; more durable restoration than a class IV resins restoration; color stability of the enamel; Positive emotional and social response from the patients for preservation of natural tooth structure.

The purpose of this article is to discuss the considerations for dental fragment reattachment technique and to report a case of tooth fragment reattachment after retrieval from the lower lip.

## CASE REPORT

**Fig. 1:**
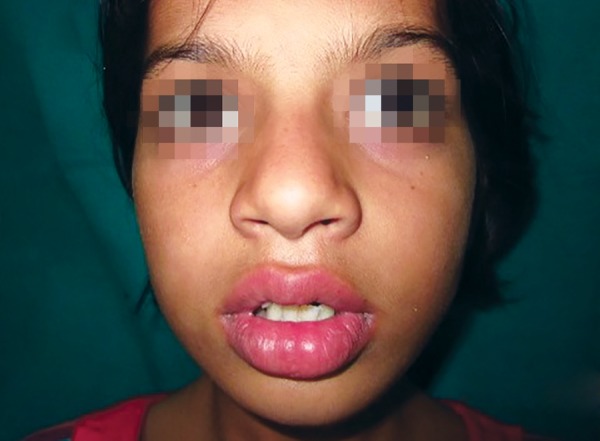
Extraoral examination showed a swelling on the left side of lip

A 12-year-old female patient reported to the Department of Pedodontics and Preventive Dentistry, SGT Dental College, following trauma to the maxillary central incisor. Trauma occurred due to fall while playing 6 months ago. Patient was attended by her general medical practitioner within 1 hour of trauma. On inspection, a swelling on the left side of lip was noticed. A firm nodule measuring approximately 1 cm in diameter in the same region was palpated (Fig. 1). Intraoral examination revealed fractured maxillary permanent central incisors.

**Fig. 2:**
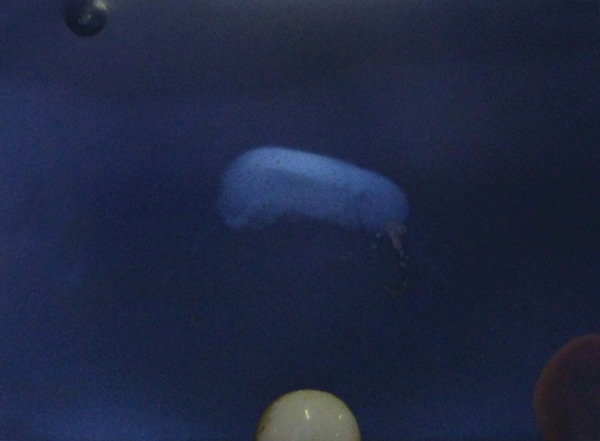
Radiograph of lip confirmed presence of tooth fragment in lower lip

No mobility of the concerned teeth was recorded and surrounding tissues were healthy. Tooth showed no vitality for pulp tests. Radiograph of the lip confirmed the presence of a tooth fragment in the lower lip (Fig. 2). The treatment plan was surgical removal of the tooth fragment from the lip and reattachment of the fragment to the tooth following root canal therapy with respect to 21 and composite build up with respect to 11.

**Fig. 3:**
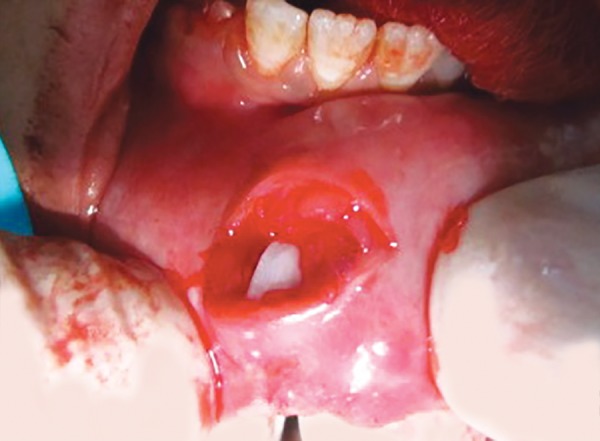
Embedded tooth fragment in lower lip

**Fig. 4:**
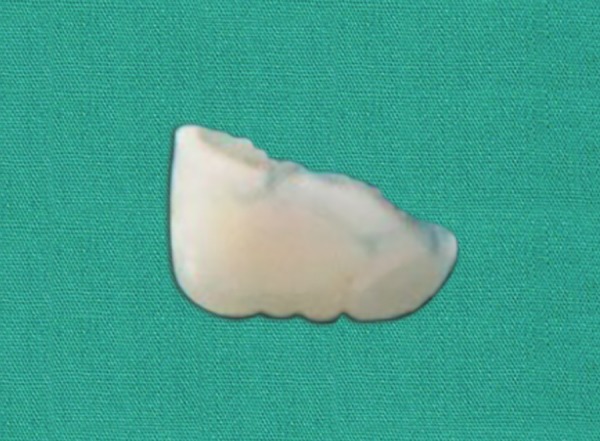
Fractured tooth fragment of 21

**Fig. 5:**
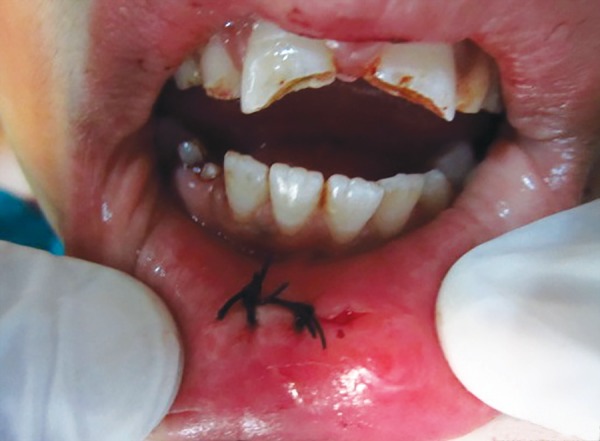
Sutures placed

*Surgical removal of the tooth fragment from the lip:* The patient was submitted to surgical excision of the fragment under local anesthesia. The lower lip was incised, tissues were reflected and the tooth fragment was located (Fig. 3). The tooth fragment was carefully removed (Fig. 4) and maintained in normal saline. Sutures were placed (Fig. 5) and patient was recalled after 7 days for the suture removal.

**Fig. 6:**
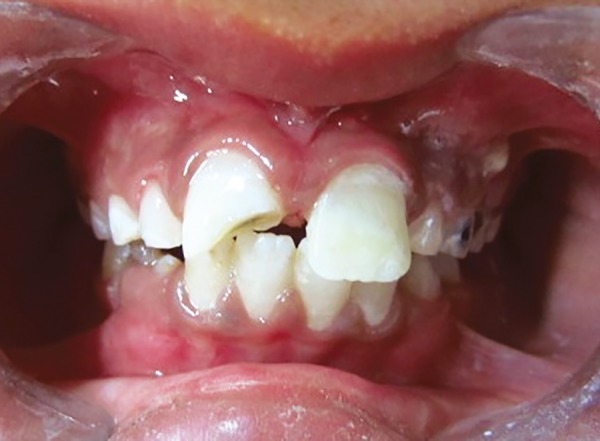
Reattached fractured fragment with respect to 21

**Fig. 7:**
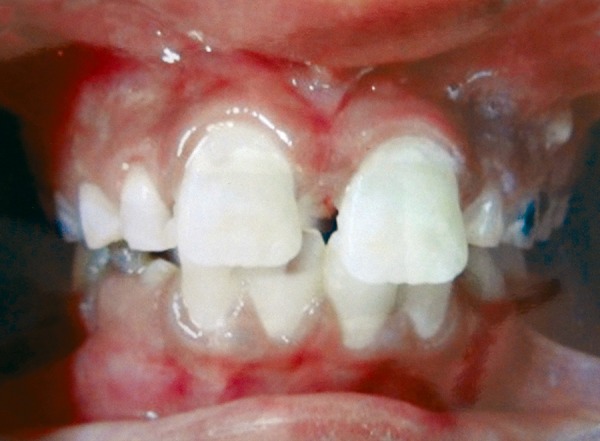
Composite build up with respect to 11

*Reattachment of the fragment to the tooth following root canal treatment:* Meanwhile, root canal therapy was completed in 21 and then the adaptation of the fragment was checked. Slight beveling of the tooth was done to increase the surface area for etching and attachment of the tooth fragment 37%. Phosphoric acid gel was applied to the enamel of the fragment and the teeth for 20 seconds. Air-water spray was used to remove the acid and the surface was air-dried. An adhesive system was applied to the tooth fragment, which was then reattached to its proper position. Visible light polymerization was done for 60 seconds to the facial and palatal surfaces of the tooth, while the fragment was kept in position under pressure. The tooth was polished with polishing disks (Figs 6 and 7).

## DISCUSSION

Dental traumas are one of the most frequent facial traumas, especially in children and adolescents. These traumas may result from various factors like falls, being the most common, followed by assaults, sports, work accidents and others.^10^

The incidence of anterior teeth crown fractures in the permanent dentition is about 26 to 76%.^11^ The upper central incisors are the teeth most frequently affected by trauma and this high incidence can be related to the anterior anatomical position and to the protrusion caused by eruptive process.^12^ Usually, a fractured or missed incisor does not pose any problem in diagnosis.^13^ However, when this situation is added to soft tissue laceration, attention should be paid to whereabouts of the teeth. The proper radiographic evaluation of the patients that missed partially or totally their teeth after maxillofacial trauma is extremely important, as long as teeth and dental structures may become foreign bodies at risk for ingestion, inclusion in surrounding tissue or aspiration. A soft tissue radiograph can well be an occlusal view or radiograph film placed between lips and dental arch with low exposure.^14^ The worst complication is aspiration of foreign bodies that can lead the patient to a variety of chronic airway problems and even death if not precociously diagnosed.^15^ Another important factor is the differential diagnosis, mainly in delayed traumas, because the radiographic image of dental fragments included in the mouth floor can be similar to sialolithiasis of the salivary glands. In the case presented here the tooth fragment was embedded in the lower lip which was confirmed by the radiograph.^13^ The remarkable advancement in adhesive systems and resin composites has provided a favorable prognosis for the reattachment of a tooth fragment.^16^ However, this technique can be used only when the intact tooth fragment is available and it should be considered as the first choice of treatment,^17^ as it offers a most functional and esthetic treatment option.^18^ As with conventional restoration, restorative success hinges on proper case selection and strict adherence to sound principles of periodontal and endodontic therapies, and the techniques and materials for modern adhesive dentistry.^19^ Diagnosis of a pulpal lesion becomes extremely important when the restoration of fractured anterior teeth is considered.^16^ The success of restorative treatment will depend on steps taken to maintain pulpal vitality. Endodontic treatment is advised in case of pulp necrosis. The reattachment of a normal tooth fragment can eliminate the problem of wear and unmatched shades associated with different restorative materials and techniques.

Reattachment techniques varied, from simple reattachment depending solely on micromechanical bonding to various preparation techniques of the tooth and the fragment. The technique that is considered more reliable is the one that provides an additional preparation, this includes enamel bevels, internal enamel or dentin grooves, chamfers and over-contouring.^20^ The research conducted by Reis et al^21^ underlined the need of executing a bevel, a chamfer or an overcontour to improve the resistance to the fracture following the bonding of a fragment. This study shows how the presence of an over contour on the fractured line related to an internal groove may increase the possibility of a tooth fracture.

Dean et al explore the influence of mode of preparation upon fracture resistance of reattached fragments.^22^ They conclude that 45° bevel does not increase tooth’s strength. Fractured teeth reattached without preliminary preparation have shown resistance as those beveled 45°. Worthington et al show similar results. In the study, they make internal and external bevels in the fragment and the remaining tooth and stand that the retentions made do not increase fracture resistance. The authors even point out that addition of resin in the bonded area do not increase fracture resistance compared to the group of teeth reattached only with bonding agent.^23^

## CONCLUSION

Tooth fragment reattachment procedure offers ultra-conservative, cost effective, safe, fast and esthetically pleasing results when fragment is available. Every attempt should be made to locate the missing tooth structure through a detailed history of the accident, careful examination and roentgenograms. The reattachment of the tooth fragment as a restorative procedure becomes possible only when it is available. This can be improved with different adhesive techniques and restorative materials.
